# An unexpected and suspended time

**DOI:** 10.1186/s10194-020-01112-7

**Published:** 2020-04-22

**Authors:** Paolo Martelletti

**Affiliations:** 1 Department of Clinical and Molecular Medicine, Sapienza University of Rome, Rome, Italy; 2 Emergency Medicine CoViD-19 Unit, Sant’Andrea University Hospital, Rome, Italy

The pandemic that is shocking our lives will also have an enduring impact on global research priorities [1–3]. It will not be a question of scotomizing important areas of medicine, but a return to its origins. The extreme fragmentation of the healthcare offers to citizens changes its priorities, and bringing together apparently distant disciplines [4]. The powerful investments that all nations are making by transforming their hospitals will not and must not be nullified but converted into large multidisciplinary departments.

In this new scenario, how will clinical and basic headache research be positioned? Will it have the same access to funds, or will global priorities decrease their force of impact, putting them at least on standby?

PubMed reports for the year 2020 729 papers published with “migraine” search, while with “CoViD-19” or “coronavirus” we can see 3746 items [5]. It is clear from now that in the next 2–3 years basic, clinical and epidemiological research on this pandemic will absorb most of the publications. Headache area will be strongly penalized, politically perceived as not a priority, previously enriched by the use of monoclonal antibodies, and not lastly for an exquisite, ethical reason.

How is the headache community going to renew itself, hooking this terrible situation? [6] To give a definite answer today is premature, but surely this pandemic will change over time the epidemiological expression of chronic non-communicable diseases. In the next 10 years we will see growing many pathologies unveiled or triggered by the immunological interactions of SARS-CoV-2 infection [7].

Therefore, epidemiological studies on CoViD-19 clinical interactions, on environmental factors, and not only on the headache symptom which sums up to 14% of CoViD-19 patients’ clinical presentations, will have to inspire the finding of still hidden avenues of research, or their *ex novo* creation.

Lastly, the huge economic crisis coming ahead will impact also on availability of funds, prioritized to the CoViD-19 area, and surely migraine won’t be top ranked in this list of priorities anymore. Which young researcher, now that hospitals are hiring all the youngsters in the CoViD-19 areas, will have scientific interest in migraine? This will depend from the vitality and creativity of our scientific community.

After the pandemic the scientific priorities will see a revolution, and this will also bring positive notes. We will see a post-crisis Marshall Plan, and it will be up to us to be part of it.

The signs of discontinuity, with the swirling chasing each other marking future appointments through the “see you at”, are clear and will remain so. No congress or meeting of medium or large size upwards will be classed as an acceptable and accepted risk. In an already digital era, Scientific Societies found themselves unprepared for remotely cultural exchanges, being anchored to a twentieth-century concept of solving problems with a handshake, or cementing ideas during the coffee breaks. The immense economic resources previously allocated to oceanic meetings, fleets of airplanes to bring together scientists, will no longer exist at least for a few years, perhaps forever. And this will mitigate the enormous conflicts of interest that undermined their credibility and therefore their reason for being.

From now on, the sharing and the scientific debate will use our digitalized desks, with the predictable reluctance of some related to habits or the fact that digital sharing prefers and rewards active participants and not mere audience. Young researchers, the sherpa of scientific research, will adapt themselves in a Darwinian way to this evolutive phase [8]. The true congresses of the future will harbour in scientific journals that will have to offer not only the aseptic list of papers already undergone the rigorous scrutiny of referees, but the critical re-reading and vulgarization of the messages contained through blogs, dedicated webinars, audio-podcasts, discussion forums, Q&A interviews, hybrid or scientific/educational platforms or new models of augmented reality interaction [9]. Is it just not the digital translation of what already took place in any conference room?

If all of us have reconverted our academic teaching from frontal to online in a flash, if we made operational methods of university exams and degrees online, then we are ready to move from the playful and social use of technology to a digital revolution where the sharing of research and the provision of training will find its territory, in emergency for now, stable immediately afterwards [10].

It is time to discover new avenues of research and education.

*Aut inveniam viam aut faciam.*

Hannibal Barca (247–182 B.C.)

## Data Availability

Not applicable.
